# Action of Iodine and Iodide of Potassium on the Nervous System

**Published:** 1865

**Authors:** 


Action of Iodine and Iodide of Potassium on the Nervous System.—The *Brit. Med. Jour.* states that Dr. M. Benedikt, having observed that the injection of tincture of iodine suddenly produced paralysis of respiration and circulation, has been led to investigate the action of iodine on the nervous system. His experiments, seventy in number, have been made on frogs. The solution of iodide of potassium used contained one part in four of water; the tincture of iodine had a strength of one part to three or six. He has found that iodine and iodide of potassiam, especially the latter, immediately affect respiration; that sensation is diminished, and finally disappears; that the heart is paralyzed more quickly by iodine than by iodide of potassium; and that muscular contractility is lost sooner than that of the heart when small doses are employed. The application of iodine or of iodide of potassium to the central extremity of the spinal cord arrests respiration, circulation, and muscular contractility much more rapidly than when the poison is introduced into the circulation. The symptoms of poisoning are more slow in appearing when the poison is applied to the peripheric extremity of the cord. Introduced into the circulatory current, iodine and iodide of potassium attack the central extremety of the cord, and excite or paralyze the organs of respiration and circulation, and the sensory and motor nerve-fibres."

